# Drug Management of Inflammatory Bowel Disease (IBD): A Narrative Review

**DOI:** 10.7759/cureus.95635

**Published:** 2025-10-28

**Authors:** Priyadarshan Singh, Paramjit Saini, Pravin P Kalyankar, Kamaljit R Kailey, Rohan Chauhan, Anupamdeep K Pannu, Navpreet Singh, Ketan Garg

**Affiliations:** 1 Internal Medicine, Cambridge Health Alliance, Cambridge, USA; 2 Internal Medicine, Christian Medical College and Hospital, Ludhiana, IND; 3 Internal Medicine, Fortis Escorts Hospital, Faridabad, IND; 4 Medicine and Surgery, Gian Sagar Medical College and Hospital, Patiala, IND; 5 Internal Medicine, Fortis Hospital, Ludhiana, IND; 6 Family and Community Medicine, Texas Tech University Health Sciences Center, Permian Basin, USA; 7 Internal Medicine, Dayanand Medical College, Ludhiana, IND; 8 Pathology, Jyoti Gupta Clinic, New Delhi, IND

**Keywords:** crohns, gastro-intestinal surgery, imunotropic drugs, inflammatory bowel syndrome, management rationale, transmural inflammation

## Abstract

The exact underlying etiopathogenesis of inflammatory bowel disease (IBD) remains unclear. Conventionally, autoimmune mechanisms have been linked with IBD, and thus various immunomodulatory or anti-inflammatory drugs have proven useful. The understanding of the complex gut mucosal immune system and the trafficking of leukocytes toward the intestine from lymphoid organs has allowed us to use molecular drugs that may target specific pathways, like the Janus kinase (JAK) pathway, phosphodiesterase, interleukins (IL)-12, 23, and adhesion molecules such as selectins. These agents have brought about a drastic change in the management strategy of IBD. Since many patients may not improve on conventional therapy, newer treatments are tried, which show better efficacy with fewer side effects. The present review highlights the concepts of newer therapeutic drugs, their interaction with host microbiome and gut-brain axis, and the new targets in the autoimmune phenomena, thereby providing a promising treatment for a better management of IBD in the present and future times.

## Introduction and background

Inflammatory bowel disease (IBD) is an autoimmune disease of the gastrointestinal tract characterized by chronic inflammation. Being idiopathic or autoimmune in nature, certain environmental and genetic factors play a role along with the gut microbiome [1].

Two primary forms of IBD have been consistently recognized, that is, Crohn's disease (CD) and ulcerative colitis (UC), with an intermediate form of indeterminate colitis. CD has been characterized pathologically by transmural inflammation involving any part of the gastrointestinal tract, whereas UC is more restricted to the colon, especially the mucosal portion of the colon [1].

The incidence of IBD has been rising worldwide, both in developed and developing countries, with adolescents and early adults being the peak age group affected. This has been linked to the inappropriate use of antibiotics for viral infections in children and adolescents, particularly early in life, which can disrupt the gut microbiome and has given rise to several hypotheses regarding autoimmune processes affecting the gastrointestinal tract [1,2].

Patients present with varied clinical features involving the symptoms related to upper and lower gastrointestinal tract such as diarrhea, early satiety, abdominal pain, rectal bleeding, weight loss, poor absorption resulting in stunting of the height, failure of the growth and systemic symptoms such as fatigue, fever, uveitis, arthralgia, ulcers in the mouth, digital clubbing and a variety of extra-intestinal manifestations which are one of the characteristics of IBD (in comparison to irritable bowel syndrome or IBS) and are more seen in young patients in about 25-35% cases [1-4]. Globally, IBS affects about 4-5% of people, while IBD affects 0.3-0.5%, yet IBD carries greater morbidity. Both share symptoms and demographics, with abdominal pain and diarrhea being the common ones. But IBD shows more severe gastrointestinal symptoms and gut inflammation, unlike IBS, which is functional, based on symptoms and ruling out other conditions and any structural damage [5].

For evaluation, standard investigation protocols have been followed, which rely on physical examination, history, human leukocyte antigen (HLA) phenotyping, and biochemical investigations for inflammation, such as complete blood counts, C-reactive protein, erythrocyte sedimentation rate, and stool examination. Radiographic evaluation has also been used in the form of computed tomography (CT) scan, magnetic resonance imaging (MRI), fluorescent scan, and endoscopic evaluation [6]. Analyzing all this, the management relies on the extent and severity of the symptoms of IBD after diagnosis.

## Review

The primary conventional treatment for IBD includes the use of corticosteroids, 5-aminosalicylates, also known as 5-ASA, and agents that suppress the immune system, known as immunosuppressive agents [7]. Infliximab is one of the most important immunomodulator therapies that acts against tumor necrosis factor alpha (TNF-α). It was one of the first immunomodulator therapies for which approval was given in the management of IBD. Following that, various other drugs that target TNF-alpha, such as adalimumab, certolizumab, and golimumab, were added to the list [2,7].

Since TNF-α is a cytokine central to the pathophysiology of IBD, and acts as a key mediator through which multiple cytokines and leukocyte trafficking are regulated, targeting TNF-α helps to interrupt the disease process rather than directly inhibiting its etiology. Infliximab, a TNF-α inhibitor, has been used for a long time; however, concerns have emerged regarding its long-term use, particularly related to its high cost, the optimal timing of initiation during the course of chronic disease, and the most appropriate dosing strategy. To address these challenges, biosimilar agents of infliximab, such as CT-P13 (marketed as Remsima® and Inflectra®), were introduced [8].

These biosimilar drugs are complicated in terms of their development, but they are made in the bodies of living organisms, such as mouse myeloma cells. The backbone is chemically made of amino acid sequences and is defined and stable. It undergoes the process of post-translational modification and glycosylation. The process is very specific to the cells in which they are produced. Batch-to-batch variations are bound to occur, but the whole process is well-regulated. Overall, the use of these drugs may bring about a shift from earlier anti-TNF-α drugs. The therapeutic landscape of IBD management is changing [7].

Another thing to discuss is the method of delivery of these drugs, which may involve the oral route, parenteral route, and rectal administration. Among them, the oral route is the most effective mode of drug delivery; however, low specificity and drug reactions at the level of systemic response have restricted the specific action at the inflamed site. To subvert this, newer drug delivery systems have come into practice, such as nano-drug delivery or enteric-coated microneedle pills, lipid-based vesicular systems, pro-drug systems (5-ASA formulations), biologic drug delivery (genetically engineered bacteria or eukaryotic cells), and hybrid drug delivery systems [9]. Besides focusing on the route of drug administration, a lot of newer drugs are being approved or are in the trial phase.

Drugs that restrict the migration of inflammatory cells

During inflammation, the T-cells are recruited from the secondary lymphoid organs to the inflammatory site of the gastrointestinal tract. Various chemokines and selectins are released, which help in the adhesion of the T-cells that have integrins on their surface. These integrins bind to the high endothelial venules (HEV) through ligands, and ultimately, they are transmigrated to the intestinal tissue [10].

**Figure 1:**
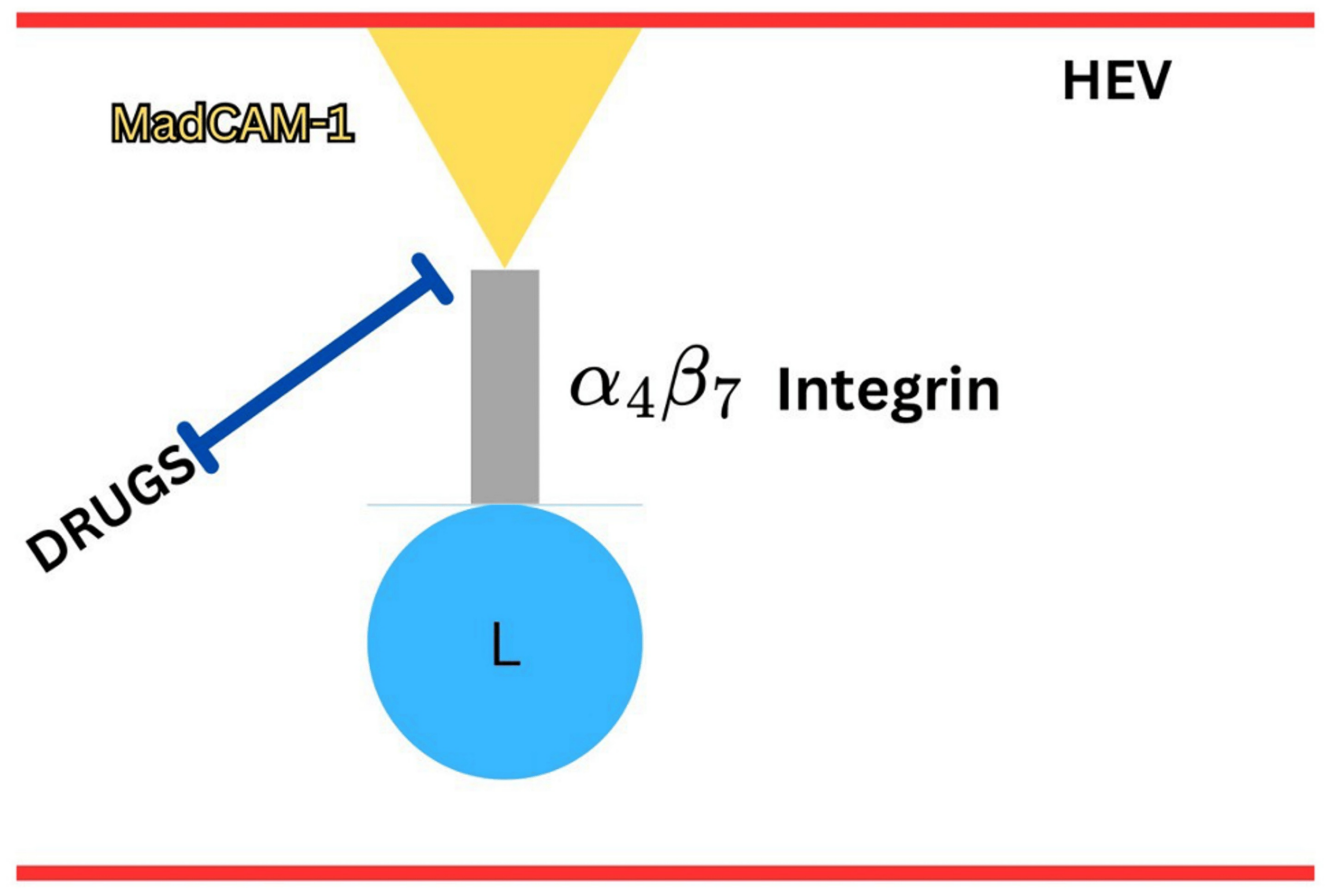
Anti-integrin drug mechanism for the management of IBD. Drugs inhibit the adhesion of integrin with MAdCAM-1 present on the high endothelial venules (HEV) and prevent emigration of lymphocytes (L) to the intestinal site IBD: inflammatory bowel disease Image created by the authors using information from Gubatan et al. [9]

The α4β7 integrin present on the lymphocytes is one of the most important antigen molecules that binds to MAdCAM-1 present on high endothelial values. Specific humanized monoclonal antibodies have been tested against subunits of the α4β7 integrin, the whole integrin, or against MAdCAM-1. These drugs are also known as anti-adhesion agents. They prevent the adhesion of lymphocytes to MAdCAM-1 present on high endothelial values and thus restrict the transmigration toward the inflammatory site [11]. The drug mechanism is shown in Figure 1.

Among all the drugs acting on integrins, vedolizumab has been approved for both CD and UC, and it acts on α4β7 integrin [12]. It has been found to be very selective and safe since it does not act or prevent CNS migration of lymphocytes in comparison to one of the drugs (i.e., natalizumab), which restricts the CNS homing of lymphocytes and thus allows for CNS infections such as progressive multifocal leukoencephalopathy, which is caused by the JC virus [13]. Among other drugs, ontamalimab is still in phase 3 for IBD [14]. Carotegrast methyl, which acts on alpha 4 integrin, has been approved for use in Japan, but it has been associated with cases of PML [13].

Beside the primary α4β7 integrin and MAdCAM-1, action on other adhesion molecules like selectins (E-, L-, P-selectin), cellular adhesion molecules (ICAM-1, ICAM-2, VCAM-1), macrophage (macrophage galactose lectin, CD44, folate receptor-beta, F4/80, mannose receptor, transferrin receptor), intestine epithelial cell (CD44, CD98, transferrin receptor), and dendritic cell (F4/80; macrophage galactose lectin, CD44, mannose receptor) has also been targeted [14,15].

Another category of drug based on the targeting molecule (sphingosine-1-phosphate receptor 1 - S1PR1), which restricts the primary emigration of lymphocytes from the lymphoid organs by internalizing the receptors and degrading them, is the S1P modulators. This receptor (S1PR) consists of five types (types 1-5), and based on the action of the drug on whichever type of S1PR, the drugs have been categorized [16]. These drugs not only allow the management of IBD but also allow the management of various other autoimmune diseases like rheumatoid arthritis, multiple sclerosis, psoriasis, systemic lupus erythematosus (SLE), atopic dermatitis (AD), UC, and CD [16].

Ozanimod is one of the S1P modulators active against one and five types of S1PR, which has been approved for UC but is still in phase 3 for CD. Other drugs in trial are etrasimod, amiselimod, KRP-203 (2-amino-2-1,3-propanediol hydrochloride), VTX 002 (OPL 002), and fingolimod [16,17]. W-061, SEW2871, DOP and THI, ABC747080 and ABC294640, and LCL351 are under preclinical studies [18-23].

Ozanimod

Ozanimod, a first-in-class S1P receptor modulator, has shown significant efficacy in treating UC [24]. Initial safety trials in healthy volunteers showed good tolerability. In the TOUCHSTONE phase 2 trial, ozanimod (1 mg/day) led to higher remission rates than placebo at weeks 8 and 32 [25]. The phase 3 TRUE NORTH study further confirmed its efficacy. Compared to placebo, ozanimod demonstrated significantly higher clinical remission rates during both induction (18.4% vs. 6%, p < 0.001) and maintenance (37% vs. 18.5%, p < 0.001) and significantly higher incidence of clinical response during induction (47.8% vs. 25.9%, p < 0.001) and maintenance (60% vs. 41%, p < 0.001). The incidence of serious infections remained below 2% across both groups [26]. Long-term data from the TOUCHSTONE-OLE study showed sustained benefits for up to four years, with 93.3% clinical response and 82.7% remission at week 200. Common adverse events (AEs) included anemia, headache, elevated liver enzymes, and mild infections [27].

Etrasimod

Etrasimod is a selective S1P receptor modulator (S1PR1, S1PR4, S1PR5) that reduces inflammation in UC by regulating immune cell migration [28]. The phase 2 OASIS trial demonstrated its efficacy, with 41.8% endoscopic improvement at 12 weeks versus 17.8% with placebo. Long-term data showed sustained clinical response (85%), remission (60%), and endoscopic improvement (69%) over 52 weeks, with mostly mild or moderate AEs [29]. The ELEVATE UC trials demonstrated superior efficacy of etrasimod over placebo in achieving clinical remission [30,31]. In ELEVATE UC 12, remission was observed in 25% of etrasimod-treated patients versus 15% on placebo (p = 0.026) [30]. In ELEVATE UC 52, 27% of patients achieved remission at week 12 versus 7% on placebo, and 32% versus 7% at week 52 (p < 0.0001) [31] and reported 32% remission vs. 7% (p < 0.0001). AEs occurred in 71% versus 56% of patients in UC 52, and 47% in both groups in UC 12. No deaths or malignancies occurred [30,31]. 

Amiselimod

Amiselimod is an oral selective S1PR1 modulator known for its favorable cardiac safety profile compared to other drugs in its class [32]. In a phase IIa trial, 0.4 mg of amiselimod was tested over 14 weeks in CD but failed to show superiority over placebo in inducing a clinical response after 12 weeks [33].

Risankizumab

Risankizumab, a selective IL-23p19 inhibitor, has shown significant efficacy in UC and CD [34-36]. The COMMAND maintenance study confirmed sustained remission with 180 mg and 360 mg doses every eight weeks [34]. In CD, the ADVANCE and MOTIVATE phase 3 trials demonstrated robust clinical remission and endoscopic responses at week 12 with risankizumab (600/1200 mg IV) versus placebo [35]. The FORTIFY study further showed long-term efficacy with subcutaneous maintenance doses [27]. Overall, risankizumab is emerging as a strong option for IBD patients, including those with previous biologic failure or complex disease history [34-36].

Mirikizumab

Mirikizumab, another IL-23p19 inhibitor, has demonstrated efficacy in both UC and CD [37,38]. In the phase 3 trial for UC, mirikizumab 300 mg IV induced clinical remission at week 12 (24.2% vs. 13.3% placebo) and maintained remission at week 52 with 200 mg SC dosing (49.9% vs. 25.1%). Extended data showed that 65.6% of patients remained in remission at week 104, with even higher rates observed in biologic-naïve patients. AEs included herpes zoster and cancers, primarily colorectal [37]. In CD, a phase 2 study showed endoscopic responses at week 12 with mirikizumab (25.8%-43.8% depending on dose), maintained through week 52 [38]. Safety was comparable to placebo, although nonrandomized maintenance patients had higher AE and discontinuation rates. Mirikizumab showed promise, particularly for biologic-refractory patients. Its gut-selective mechanism and sustained symptom control make it a promising candidate for chronic IBD management [38].

Guselkumab

Guselkumab, an IL-23p19 inhibitor, has shown promising efficacy and safety in moderate to severe IBD [39,40]. In CD, the phase 2 GALAXI-1 trial demonstrated significantly greater reductions in CDAI and higher clinical remission rates at week 12 with guselkumab (up to 57.4%) versus placebo (16.4%). A 48-week extension confirmed sustained clinical (up to 73%) and endoscopic (up to 33%) remission, with a favorable safety profile, no deaths, opportunistic infections, or tuberculosis [39]. In UC, the phase 2b QUASAR trial showed significantly higher clinical response rates at week 12 with guselkumab 200 or 400 mg (61.4% and 60.7%) compared to placebo (27.6%, p < 0.001). Serious AEs and treatment discontinuations were lower in guselkumab groups, and no deaths or serious infections occurred [40].

Brazikumab

Brazikumab, an IL-23p19 monoclonal antibody, is a potential therapeutic candidate for moderate to severe CD, particularly in patients with prior biologic failure [41]. In a phase 2a randomized controlled trial, patients received 700 mg IV at weeks 0 and 4, followed by 210 mg subcutaneously every four weeks from week 12. Clinical response at week 8 was significantly higher with brazikumab (49.2%) versus placebo (26.7%), and endoscopic response at week 12 was 27.1% compared to 6.3% in placebo. An open-label extension showed 63.4% maintained clinical remission and 31.7% sustained endoscopic response at week 56. In a longer-term follow-up, 54.8% completed 100 weeks of therapy. Discontinuation was mainly due to lack of efficacy or adverse effects. AEs were mostly mild to moderate, including headache, nasopharyngitis, and upper respiratory infections. No deaths, malignancies, or serious opportunistic infections were observed. These findings support brazikumab’s durability and acceptable safety profile in refractory CD management [42].

Cytokine inhibition

Cytokines have been targeted at multiple steps. The primary targets have been interleukin (IL)-12 and IL-23, which are released from myeloid cells and are involved in the differentiation of naïve CD4-T cells into helper T-cells TH1 and TH17. These helper T-cells are involved in the pathogenesis of IBD, and the drugs have been targeting units of IL-12 or IL-23, primarily the P40 subunit or the P19 subunit of IL-23 [43].

IL-12 is composed of a heterodimer of two subunits, P35 and P40, while IL-23 is composed of two subunits, P19 and P40. P40 is a common subunit of IL-12 and IL-23. Ustekinumab, which is a human monoclonal antibody, is an antibody to the P40 subunit, and it basically blocks the action of both IL-12 and IL-23. This is one of the drugs consistently used for IBD, which is approved for both UC and CD. Apart from that, four other drugs, brazikumab, risankizumab, guselkumab, and mirikizumab target P19 subunits. Among these, only risankizumab is used for CD, while others are in trial phases. Action on only the P19 subunit of IL-23 allows them greater affinity, thereby limiting their side effects. The side effects, which are mainly prevented by these drugs in comparison to ustekinumab, are the IL-12-mediated activation of T-cells involved in managing infections and immunity to cancer cells [2,43].

Another pathway that has been targeted is the Janus kinase (JAK)/signal transducer and activator of transcription (STAT) pathway, wherein cytokines are blocked that are involved in the induction of JAK or the transduction of signals through JAK. JAK is a tyrosine protein kinase family member having four members, JAK1-3, and tyrosine kinase 2, also known as TYK2. Oral drugs are used to inhibit the JAK signaling pathway, allowing for a quick action and fast relief from symptoms of IBD. Multiple cytokines that are involved in different members of the JAK stat kinase pathway are targeted, and thus, a wider response is observed [2,43].

Of the various drugs, tofacitinib acts nonselectively on the JAK/STAT kinase pathway, while others like filgotinib are JAK-1-specific. Upadacitinib is JAK-1 specific, while deucravacitinib is TYK2-specific. Except for deucravacitinib, which is in trial phases, others have been approved for UC, but not for CD yet [2,43]. The ongoing trials for various JAK inhibitors include SELECTION trial, FITZROY study, U-ACHIEVE study, and CELEST trial [2,44-47].

TL1A inhibitor

Genome-wide association studies have identified TNFSF15, also known as TNF-like ligand 1A (TL1A), as a key genetic variant associated with IBD. TL1A is secreted by antigen-presenting cells upon activation and binds to death receptor 3 (DR3), playing a crucial role in both innate and adaptive immune responses. The TL1A-DR3 pathway is linked to proinflammatory effects, including T cell activation and fibrotic pathways, which are important in IBD pathogenesis. An anti-TL1A antibody, PF-06480605, has been shown to reduce Th1 and Th17 cytokine responses in tissues, leading to significant endoscopic improvement in moderate to severe UC [48].

IL-36 inhibitors

In IBD, the IL-36 family’s expression is elevated during gut inflammation. Full-length IL-36α and IL-36γ are released by various gut cells, including epithelial cells, lymphocytes, and macrophages, and are activated by neutrophil proteases. These active forms bind to the IL-36R complex, triggering proinflammatory responses in cells like dendritic cells, macrophages, and T lymphocytes. Spesolimab, a humanized monoclonal antibody targeting IL-36R, is being explored as a potential IBD therapy in clinical trials [49].

Phosphodiesterase (PDE) inhibitors

PDEs are a diverse group of enzymes responsible for breaking down cAMP and cGMP. PDE4, in particular, is present in dendritic cells, macrophages, monocytes, and T cells. Blocking PDE4 raises intracellular cAMP levels, which decreases the production of inflammatory cytokines like TNF-α, IL-17, interferon gamma (IFN-γ), and IL-23 while boosting the levels of regulatory cytokines such as IL-10 [2,50,51].

IL-10 is a key anti-inflammatory cytokine in IBD. By activating the JAK1-TYK2-STAT3 pathway in macrophages and dendritic cells, it suppresses pro-inflammatory cytokines (TNF-α, IL-6, IL-12, IL-23) and limits Th1/Th17 responses. IL-10 also promotes regulatory T cell activity, supporting tolerance to gut microbiota. Defective IL-10 signaling results in uncontrolled intestinal inflammation and IBD progression [50]. Consequently, the PDE4 inhibitor apremilast has shown effectiveness as a treatment for active UC [2,51]. 

Future perspectives

IBD, being an autoimmune disease involving multiple inflammatory pathways, has prompted continual efforts to devise newer management methods. Apart from the monotherapies, which are targeted against specific pathways, research continues to modulate the gut microbiome [52,53].

The immune system of the host and its interaction with the epithelial cells of the intestine, along with the microbiological organisms already present in the gut, has been extensively explored. The diversity of microbiota in the human gut is critical for human health, and any disturbance can lead to a variety of diseases. Patients with IBD have lower biodiversity as compared to the normal healthy population, which is a primary contributing factor to IBD. Thus, the therapeutic approach rests on modulating this microbiome. These include the use of prebiotics, probiotics, transplantation of fetal microbiota, use of synthetic bacteria, and regulation of biliary acids. These mechanisms allow for restoring the balance between immune cells and the resident gut microbiome [52-55].

## Conclusions

IBD remains a complex, multifactorial condition with rising global prevalence and significant morbidity. Advances in understanding immunopathogenesis have driven the development of novel biologics and small molecules, offering improved efficacy and safety compared to conventional therapies. Despite these advances, challenges remain regarding optimal treatment sequencing, long-term safety, cost, and accessibility. Future strategies are recommended to integrate personalized medicine, microbiome modulation, and innovative drug delivery systems to optimize outcomes. Continued research and collaborative efforts are essential to translate these emerging therapies into sustainable, patient-centered care.
